# Interannual and seasonal variations in carbon exchanges over an alpine meadow in the northeastern edge of the Qinghai-Tibet Plateau, China

**DOI:** 10.1371/journal.pone.0228470

**Published:** 2020-02-11

**Authors:** Jinkui Wu, Hao Wu, Yongjian Ding, Jia Qin, Hongyuan Li, Shiwei Liu, Di Zeng

**Affiliations:** 1 Key Laboratory of Ecological Hydrology and Basin Sciences in Northwest Institute of Eco-Environment and Resources, Chinese Academy of Sciences, Lanzhou, China; 2 State Key Laboratory of Cryospheric Science, Northwest Institute of Eco-Environment and Resources, Chinese Academy of Sciences, Lanzhou, Gansu, China; 3 College of hydraulic science and engineering, Yangzhou University, Yangzhou, China; Tennessee State University, UNITED STATES

## Abstract

The alpine meadow is highly sensitive to global climate change due to its high elevation and cold environment. To understand the dynamics of ecosystem carbon cycling, CO_2_ fluxes were measured over the Suli alpine meadow, which is located at the upper reach of the Shule River basin at the northeastern edge of the Qinghai-Tibet Plateau (QTP), China. The measurements were taken from October 2008 to September 2012 using the eddy covariance technique. Obvious seasonal and inter-annual variations were observed in the CO_2_ flux. The annual net carbon exchange ranged from -195.28 g·CO_2_·m^-2^ to -118.49 g·CO_2_·m^-2^, indicating that the alpine meadow ecosystem in this area played a role as a carbon sink. The inter-annual variability in the net carbon exchange was significantly related to the length of the growing season for the alpine meadow. The results showed that the months of June, July and August were the strongest CO_2_ absorption periods, while April, May and October were the strongest CO_2_ release periods. The annual net exchanges of CO_2_ in the four years were -118.49 g·CO_2_·m^-2^, -130.75 g·CO_2_·m^-2^, -195.83 g·CO_2_·m^-2^ and -160.65 g·CO_2_·m^-2^, and the average value was -151.43 g·CO_2_·m^-2^. On a seasonal scale, the monthly CO_2_ fluxes were largely controlled by temperature. At the annual scale, there was no dominant factor that influenced the interannual variations in the CO_2_ flux.

## Introduction

Accurate evaluations of the carbon dioxide (CO_2_) flux in different ecosystems can help deepen and improve our understanding of land-atmosphere interactions and enhance the decision-making abilities of climate policymakers [1, 2]. The evaluation of the global CO_2_ flux in an ecosystem requires the construction and validation of carbon cycle models in the major ecosystems in the word and accurate measurements of the CO_2_ flux [3]. Therefore, national and international research programs have been carried out to acquire, process and analyse the CO_2_ fluxes in major ecosystems [3, 4].

Grasslands are one of the most widely distributed terrestrial ecosystems worldwide. Natural grasslands cover approximately 30% of the Earth’s surface and contain approximately 25% of the Earth’s total amount of organic carbon [5]. Studies on the carbon exchange processes in grassland ecosystems have been conducted in Mediterranean grasslands [6], tall grass prairie [7], tropical savanna [8], alpine meadow [9] and even intensively managed grasslands [10] or degraded grasslands [11]. Whether these sites function as CO_2_ sources or sinks is still unknown [4, 12, 13]. At the ecosystem scale, grasslands may act as a net carbon source or sink or may transition between the two types [12–15]. It was reported that the carbon storage in grasslands in China was 45.51 Pg C (including the carbon stored in vegetation and soil), which accounted for 40.8% of the total ecosystem carbon storage in China [16]. The studies indicate that the grassland ecosystems in China function as CO_2_ sinks overall; however, these grassland ecosystems sometimes alternate as carbon sources due to the variations in interannual environmental conditions [9, 11, 17–23].

The key factor that controls the CO_2_ exchange in grassland ecosystems is still under discussion. Grasslands are widely distributed in regions where the annual precipitation may vary from 150 to 1200 mm. Some studies [14, 24–26] have shown that precipitation is the major factor limiting CO_2_ exchange in grassland ecosystems because the annual productivity of grasslands increases linearly with increasing annual precipitation, while other studies [6] have indicated that the length of the growing season controls the CO_2_ exchange in these ecosystems. However, a 3-year study [9] on the daily CO_2_ exchange demonstrated that the air temperature, rather than the precipitation or the length of the growing season, was the major factor limiting CO_2_ exchange in an alpine meadow.

Quantifying how the photosynthesis and respiration of vegetation respond to fluctuations in the environment and physiological variables at different time scales, the emergent scale properties were discovered due to the continuous observed carbon fluxes of ecosystems [12]. To assess annual changes in CO_2_ fluxes, datasets that have been gathered over longer durations are needed [4]. Most long CO_2_ flux studies have focused on forest [3, 27–30], grassland and cropland [11]. The AmeriFlux network, which consists of eddy covariance flux towers encompassing a large range of climate and biome types, provides the longest, most extensive, and most reliable measurements of plot-scale net carbon exchange for the U.S. [2].

Most studies on the carbon budget of grasslands have been conducted at elevations approximately at or below 1500 m [9]. Little information on carbon flux is known at higher elevations. The Qinghai-Tibet Plateau (QTP) in western China has an average elevation exceeding 4500 m, and it has an area of 2,500,000 km^2^, making it the world’s highest and largest plateau [31]. Climate change in the QTP has aroused great interest among scientists in many fields. Alpine meadow ecosystems in China, most of them distributed in the QTP, cover approximately 6.37×10^5^ km^2^ and contain 11.3 Pg of carbon in biomass and soil [32]. The soil carbon density of this ecosystem (18.2 kg m^−2^; Ni, 2002) is much higher than that of savanna [5] and temperate grassland [5] and is similar to that of tundra [1], where the low temperature limits the growing season.

In this paper, we report a 4-year study of CO_2_ exchange based on observational results over alpine meadow sites in the northwestern QTP. The purposes of this study are as follows: 1) to assess the annual change in the net ecosystem exchange (NEE) at the alpine meadow site in the QTP and to identify the possible sources of the variation; 2) to investigate the impact of the growing season length on the annual NEE; and 3) to explore how changes in gross primary production (GPP) and ecosystem respiration (R_eco_) relate to variability in the annual NEE (particularly through responses to meteorological elements, such as temperature, radiation and precipitation) and the correlation between the annual GPP, R_eco_ and NEE. We hope our study will enhance our understanding of CO_2_ exchange in alpine meadows at high elevations and provide possibilities for comparing CO_2_ exchanges with those in other regions.

## Site description and measurements

### Site description

The experiment was conducted in an alpine meadow at the Suli Ecological and Environment Station (SEES, 38°25'N, 98°19'E). The SEES is located in a large valley oriented from southeast to northwest and is surrounded by the Qilian Mountains at the northeastern edge of the QTP [31]. The study area has low air temperature, little precipitation and high evaporation and is situated in a typical continental arid desert climate region [33]. The mean annual temperature is approximately -5°C. The annual precipitation ranges from 100 mm to 350 mm, while the annual potential evaporation is approximately 1200 mm. The precipitation during the growing season from May to September accounts for 80% of the annual total precipitation.

The landscape is characterized by large mountain ranges with steep valleys and gorges interspersed with relatively level and wide inter-mountain grassland basins [34]. Frigid calcic soils and bog soils are widely distributed throughout this area [35]. The surface soil is Mat Cry-gelic Cambisols [31].

The plant community in the alpine meadow is dominated by *Kobresia pygmaea* and *Carex moocrofii* [36]. The plants begin growing in May, when the daily air temperatures are above 0°C and continue to rise. The maximum aboveground biomass is reached in July or August, which is also when the air temperature and precipitation reach their highest values of the year.

### Measurements

A 10 m tall tower with eddy covariance systems was established to measure mass and energy fluxes in the valley basin in 2008. The valley provided a uniform land cover and sufficient upwind fetch for data measurements.

#### Flux measurements

The eddy covariance (EC) system with a 3-D ultrasonic anemometer–thermometer (CSAT-3, Campbell Scientific Inc., Logan, UT, USA) and an open-path infrared gas (CO_2_/H_2_O) analyzer (IRGA) (LI-7500, LI-COR Inc., Lincoln, NE, USA) was installed at a height of 3.0 m and was used to measure energy and CO_2_ fluxes. The signals were recorded at 10 Hz by a data logger (CR3000, Campbell Scientific Inc., Logan, UT, USA).

#### Meteorological measurements

A four-component net radiometer (CNR-1, Kipp & Zonen, Delft, Netherlands) was set up at a height of 2.0 m above the ground. The air temperature (*T*_a_) and relative humidity (*RH*) (HMP45C, Vaisala, Helsinki, Finland) were measured at 2.0 m, 5.0 m, 7.5 m and 10.0 m. Based on the *T*_a_, the length of the growing season was calculated by the air temperature at 2 m, and the length of the growing season represents the period when the daily *T*_a_ was above 5 °C for at least five consecutive days [15]. To meet the need to measure the wind direction and horizontal wind velocity, windset sensors (020C-1, Met One Inc., OR, USA) were set up at the same four heights.

The thermistors (107L, Campbell Scientific Inc., Logan, UT, USA) were set to measure the soil temperature (*T*_s_) at six depths (0.05 m, 0.10 m, 0.30 m, 0.50 m and 0.70 m). The soil water content (SWC) at five depths (0.10 m, 0.20 m, 0.40 m, 0.60 m and 0.80 m) was obtained by time domain reflectometry (TDR) probes (EnviroSMART, Campbell Scientific Inc., Logan, UT, USA). Two soil heat flux plates (HFP01, Hukeflux Inc., Delft, Netherlands) were set to measure the soil heat flux in separate locations at 0.05 m and 0.10 m below the soil surface. An all-weather precipitation gauge (Geonor T-200B, Norway) was used to measure precipitation. All meteorological data were recorded every 600 s and were logged by a data logger (CR1000, Campbell Scientific Inc., Logan, UT, USA).

## Methods and data

### Methods

The NEE was computed using the observed CO_2_ concentration data acquired by the EC system [12]. The NEE was partitioned into GPP and R_eco_. To test the hypothesis that stated the relationship between the soil temperature and NEE at nighttime remains unchanged in the daytime, the daytime R_eco_ was estimated [6]. The GPP was calculated by subtracting R_eco_ from the NEE. The annual GPP, R_eco_, and NEE were integrated from 1 October of one year to 30 September of the next year to span the hydrological year. Negative NEE values represented CO_2_ uptake by the ecosystem, while positive NEE values represented CO_2_ release from the ecosystem.

### Flux data processing

In this study, Edire software was used in raw data processing. Data processing is mainly referenced by the EC data processing method recommended by FLUX NET [37]. Before the scalar flux computation, spike detection, removal and coordinate rotation were performed [31]. In addition, the sonic temperature fluctuations and Webb-Pearman-Leuning (WPL) were taken into account to correct the fluxes of sensible heat and latent heat.

Precipitation and atmospheric conditions had negative effects on the data that were measured by EC. These erroneous data were discarded from our analyses, and we used gap-filled CO_2_ flux data, as described by Falge, Baldocchi (7). For this study, instrumental error, power cuts and precipitation caused 28.2% of the missing data from the study period. Measurements taken under low turbulent mixing conditions (friction velocity < 0.16 m s^-1^) resulted in another 13.5% of missing data. Altogether, we discarded approximately 41.7% of the total data recorded over the 4 years.

Gap-filling methods have been proposed and verified by previous studies [7, 38, 39]. For this study, these gaps were filled following the strategies described by Falge, Baldocchi (7):

1. Linear interpolation was used to fill the gaps that were less than 2 h by calculating an average of the values immediately before and after the data gap. The value of 1 W· m^-2^ of total solar radiation was used as the limit for day and night [40], and we divided a day into daytime and nighttime. The missing NEE during the daytime in the growing season (from May to September) was calculated as follows:
  $$NEE=\frac{{\text{a}}_{1}\times PPFD}{a_{2}+PPFD}+a_{3}$$ (1)
  where *PPFD* is the photosynthetic photon flux density and the calculation can be referenced by the climatological method of photosynthetically active quantum proposed by Zhang, Zhang [41], and a_1_, a_2_, and a_3_ are fitting constants.
2. The missing nighttime ecosystem respiration (R_eco_) was determined from the nighttime NEE and soil temperature at a depth of 5 cm (T_s5_). We assumed that the response of R_eco_ to T_s5_ in the daytime was the same as that in the nighttime; thus, the nighttime relationship was extrapolated to estimate the daytime R_eco_. The nighttime CO_2_ flux in the growing season (from May to September) and in a day in the non-growing season (October to April) was interpolated using the following equation:
  $$R_{\text{e}co}=b_{0}\text{exp}\left(bT_{s5}\right)$$ (2)
  where *T*_s5_ is the ground temperature at 5 cm, and b_0_ and b are fitting constants.

### Statistical analysis

The relationships of GPP, R_eco_, and NEE with *T*_a_, PPT, VPD, SWC and PAR were investigated by linear regression analysis, partial correlation analysis and stepwise multiple regression analysis using monthly data in SPSS (Version 20.0, SPSS Inc., IL, USA). The statistical information for the relationships between monthly GPP, R_eco_, and NEE and *T*_a_, PPT, VPD, SWC and PAR is shown in Tables 3, 4 and 5.

## Results

### Patterns of variation in climate factors

**Fig 1:**
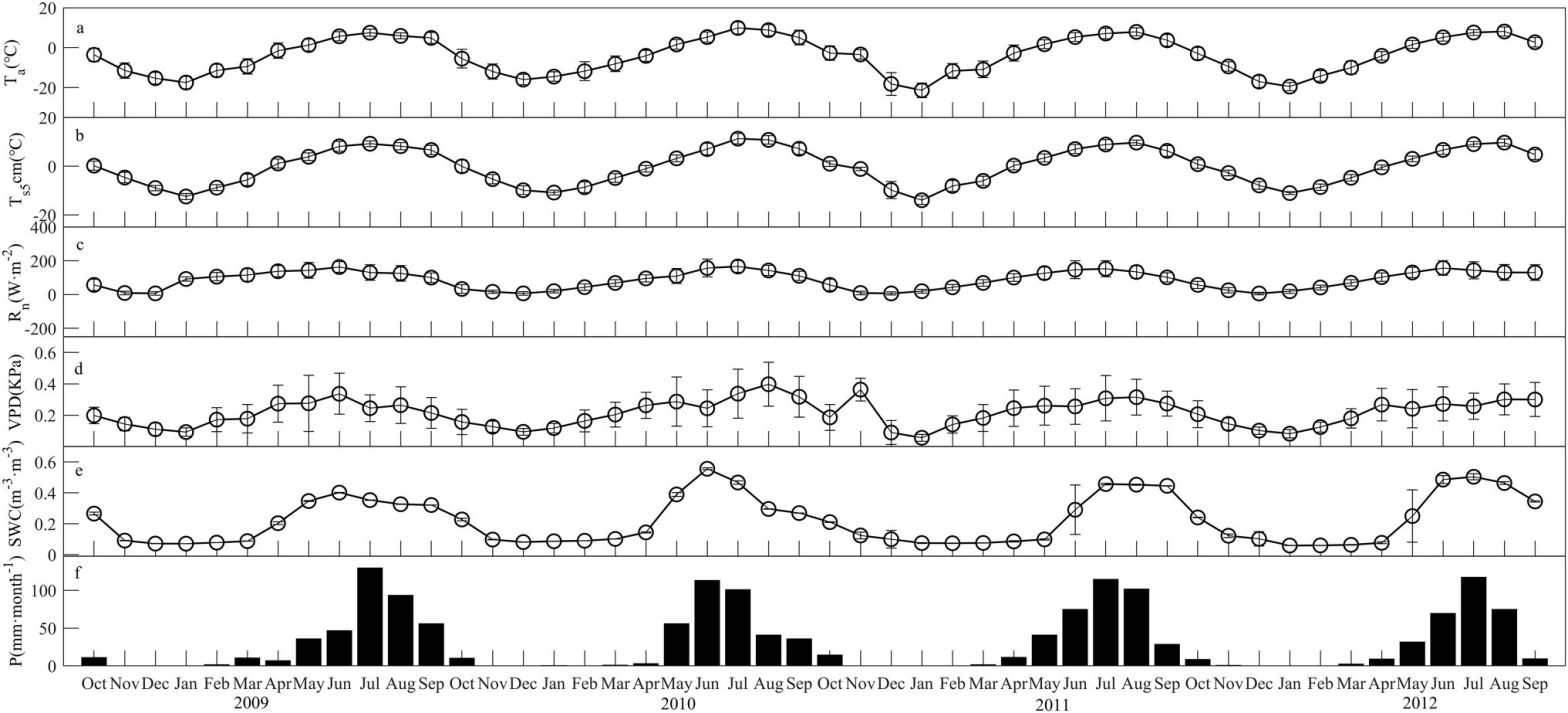
Monthly mean climate parameters. (a) air temperature (T_a_), (b) soil temperature at a depth of 5 cm (T_s5_), (c) net radiation (R_n_), (d) vapor pressure deficit (VPD), (e) soil water content (SWC) at a depth of 20 cm, and (f) precipitation for each month between 2009 and 2012. Error bars represent standard deviations of the mean.

Previous studies have not characterized the change in climate in this alpine region. To gain an understanding of the climate background in this area, we focused on the long-term variability of these climate factors by using monthly means derived from our observations. The mean annual air temperature was -3.8°C (Table 1). There were obvious seasonal changes in the monthly mean air temperature, ranging from -21.4 to 9.9°C (Fig 1a). The T_s5_, i.e., the soil temperature at a depth of 5 cm, showed a similar seasonal pattern to that of the air temperature but had a lower amplitude of oscillation; the annual mean was -0.2°C, and the range was from -14.0 to 11.3°C (Fig 1b). The daily soil temperature was positive from mid-April to mid-Oct and negative the rest of the time. There were also seasonal changes in the monthly mean net radiation (R_n_), vapor pressure deficit (VPD) and soil moisture content (SWC), ranging from 6.2 to 166.1 W·m^-2^, 0.06 to 0.40 KPa and 0.06 to 0.56 m^-3^·m^-3^, respectively (Fig 1c, 1d and 1e). The annual mean values were 86.2 W·m^-2^, 0.22 KPa and 0.22 m^-3^·m^-3^, respectively. The mean annual precipitation for the study period was 364.8 mm (Fig 1f). Previous studies showed that 90% of the precipitation was concentrated within the short growing season between May and September [33–36]. During this period, the climate conditions, including solar radiation, temperature, and water availability, are favourable for plant growth.

**Table 1:** Annual and seasonal mean air temperature, precipitation, soil temperature, and net ecosystem exchange of CO_2_ (NEE) over the alpine meadow.

Hydrological year^a^	Air temperature/°C			Precipitation/mm			Soil temperature/°C			NEE/ g·CO_2_·m^-2^· yr^-1^		
	Annual	GS^b^	NGS^b^	Annual	GS^b^	NGS^b^	Annual	GS^b^	NGS^b^	Annual	GS^b^	NGS^b^
2008–2009	-3.7	5.1	-10.0	390.1	360.4	29.7	-0.3	7.2	-5.6	-118.49	-312.31	193.82
2009–2010	-3.4	6.1	-10.3	360.1	345.7	14.4	-0.1	7.9	-5.8	-130.75	-299.49	168.74
2010–2011	-3.8	5.1	-10.1	386.6	359.7	26.9	-0.2	7.1	-5.4	-195.83	-376.51	180.68
2011–2012	-4.3	5.0	-11.0	322.5	301.8	20.7	-0.1	6.6	-5.0	-160.65	-313.72	153.07
Mean	-3.8	5.3	-10.4	364.8	341.9	22.9	-0.2	7.2	-5.5	-151.43	-325.51	174.08

^a^ Hydrological year starts on 1 October and ends on 30 September of the next year.

^b^ GS refers to the growing season, from May to September in this study; NGS refers to the non-growing season, from October to April of the next year.

### Inter-annual and seasonal variability of NEE

The annual NEE of the alpine meadow ecosystem, which ranged from -195.28 g·CO_2_·m^-2^ yr^-1^ to -118.49 g·CO_2_·m^-2^ yr^-1^, fluctuated from year to year (Table 1). The ranges of the NEE in the growing season and non-growing season were -376.51~-312.31 g·CO_2_·m^-2^ yr^-1^ and 153.07~193.82 g·CO_2_·m^-2^ yr^-1^, respectively (Table 1).

On average, the alpine meadow was a carbon sink in the growing season and a carbon source in the non-growing season. The intensity of the carbon sink was greater than that of the carbon source. The alpine meadow was a carbon sink at the annual time scale. The standard deviation of the annual NEE was approximately 34.49 g·CO_2_·m^-2^ yr^-1^ for the grassland (Table 1).

**Fig 2:**
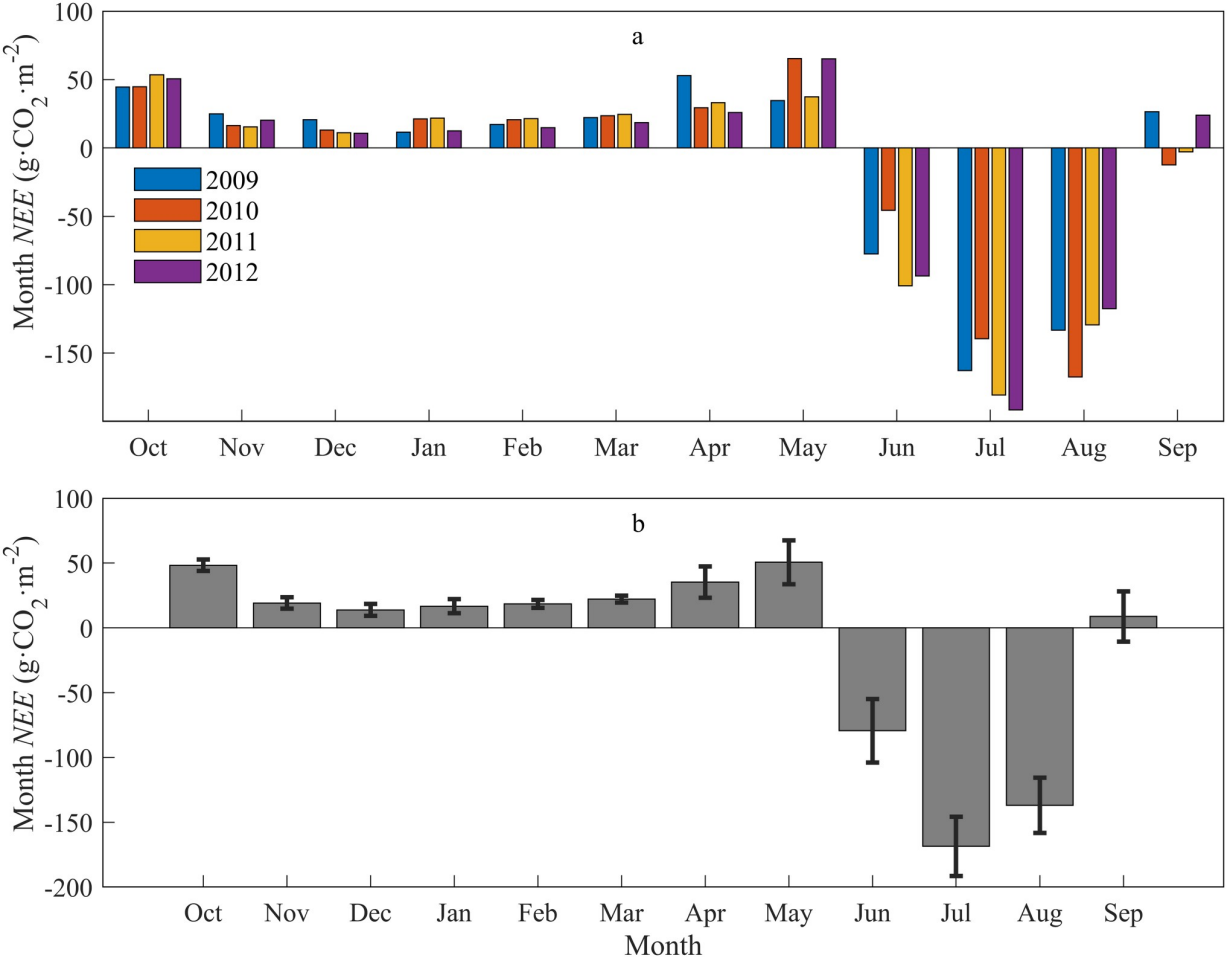
Variation in monthly net ecosystem exchange (NEE) (a) and mean monthly net ecosystem exchange (NEE) with standard deviation (b) between 2008 and 2012.

There was a clear seasonal variation in the NEE in the alpine meadow in the upper reach of the Shule River basin (Fig 2b). At the beginning of the year, i.e., from January to March, the CO_2_ release remained stable at a low level in the non-growing season. In April, the NEE increased and reached a release peak in May. The NEE turned negative in June and reached an uptake peak in July in the main growing season, while it became positive in September and reached another release peak in October in the non-growing season.

From the 2009, 2010, 2011 and 2012 observational data (Fig 2a), within four years, the same trends were basically found in the same months for the NEE. Because of different weather conditions found in each month in the different years, the NEE values were different in the same month of different years. In June, July and August, the grassland was carbon sink. In September, the grassland was mixed carbon sequestration and carbon source in different years. In the other months, the grassland served as a carbon source. The NEE inter-annual changes were largest in April, May, June and September. The maximum monthly CO_2_ absorption appeared in July 2012, and the minimum monthly CO_2_ uptake appeared in September 2011; the maximum CO_2_ emissions occurred in January and May 2010, and the monthly minimum CO_2_ emissions occurred in December 2012. The NEE had large inter-annual variability; the values from 2009 to 2012 were as follows: NEE: -118.49 g·CO_2_·m^-2^ yr^-1^, -130.75 g·CO_2_·m^-2^ yr^-1^, -195.83 g·CO_2_·m^-2^ yr^-1^ and -160.65 g·CO_2_·m^-2^ yr^-1^, respectively, with an average of -151.43 g·CO_2_·m^-2^ yr^-1^.

### Length of growing season

**Fig 3:**
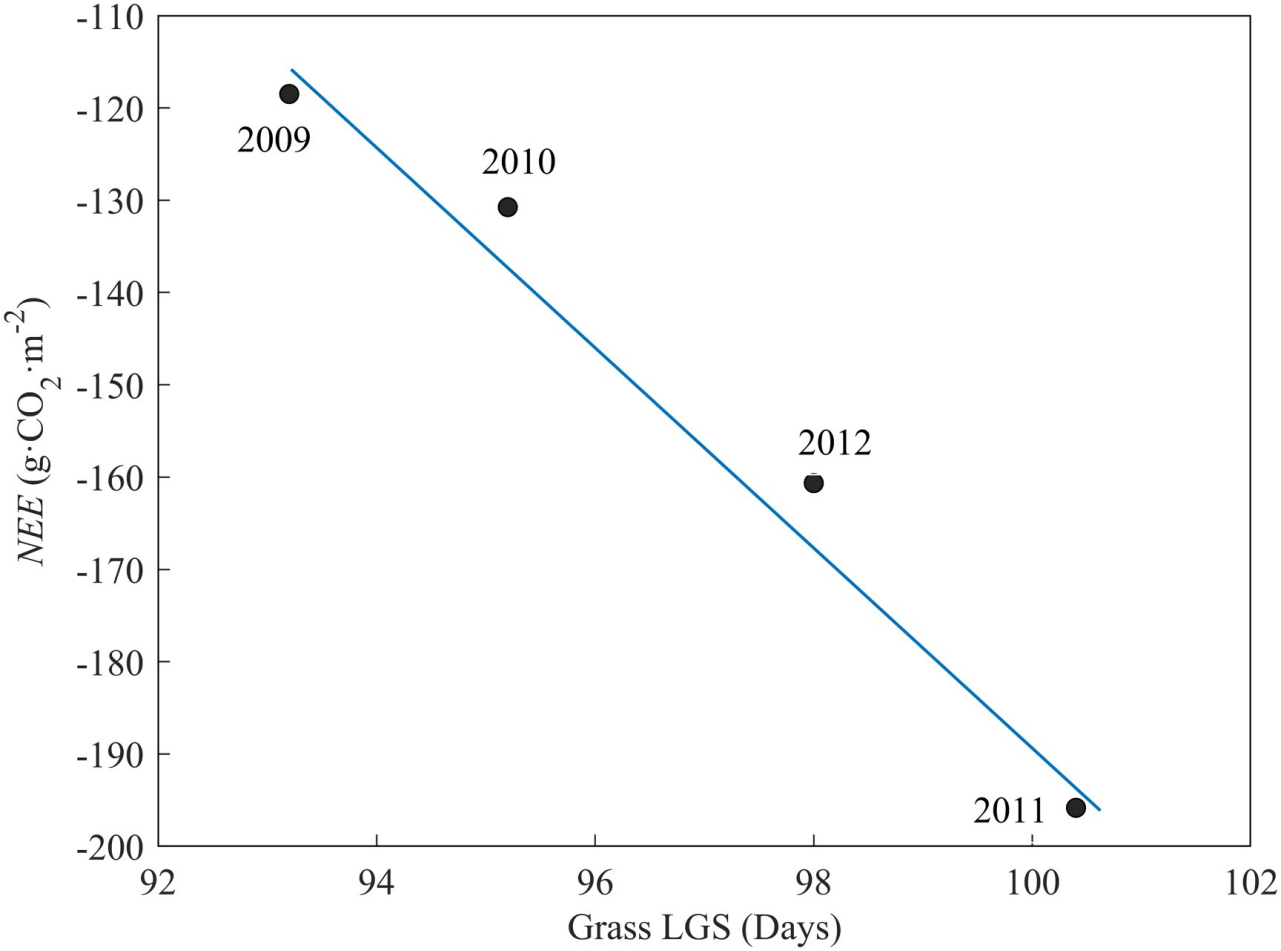
Relationships between annual NEE and length of growing season (LGS) in the alpine meadow.

The length of the growing season varied each year in the alpine meadow. The average length of the growing season was 102 days, and the standard deviation of the growing season length was 8 days. The growing season length showed a certain relationship with the annual NEE of the alpine meadow. There was a significant linear relationship between the growing season length and the annual NEE in the alpine meadow (P <0.01, R^2^ = 0.98) (Fig 3).

**Fig 4:**
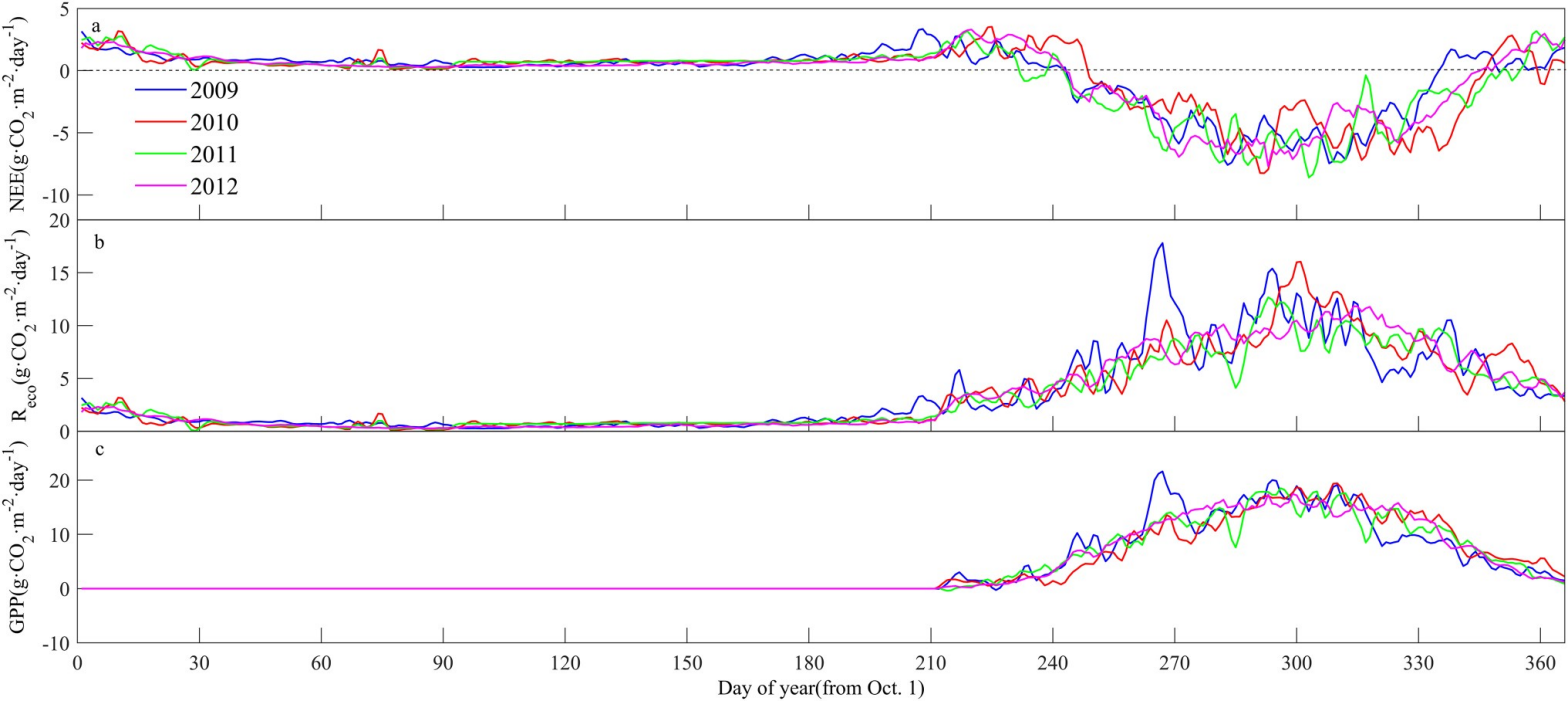
Seasonal patterns of net ecosystem exchange of carbon (NEE), gross primary productivity (GPP), and ecosystem respiration (R_eco_) in the alpine meadow.

To further understand the mechanism of the growing season length and the annual NEE in the alpine meadow, we examined the changes in the daily NEE, GPP, and R_eco_. In early May, grass had germinated. During this period, the rates of grass photosynthesis were lower and that of respiration, resulting in a positive NEE. The grassland started to take up net carbon at the end of May or in early June. The exact times were slightly different from 2009 to 2012, with dates of June 2, June 6, May 20 and May 31, respectively. The NEE, R_eco_ and GPP reached their maximum values in late July and early August. Grasses died in late September and early October, and the GPP decreased gradually to 0. The R_eco_ also decreased gradually. The grassland started to display a net carbon release, which was due to the respiration of soil microbes (Fig 4).

**Fig 5:**
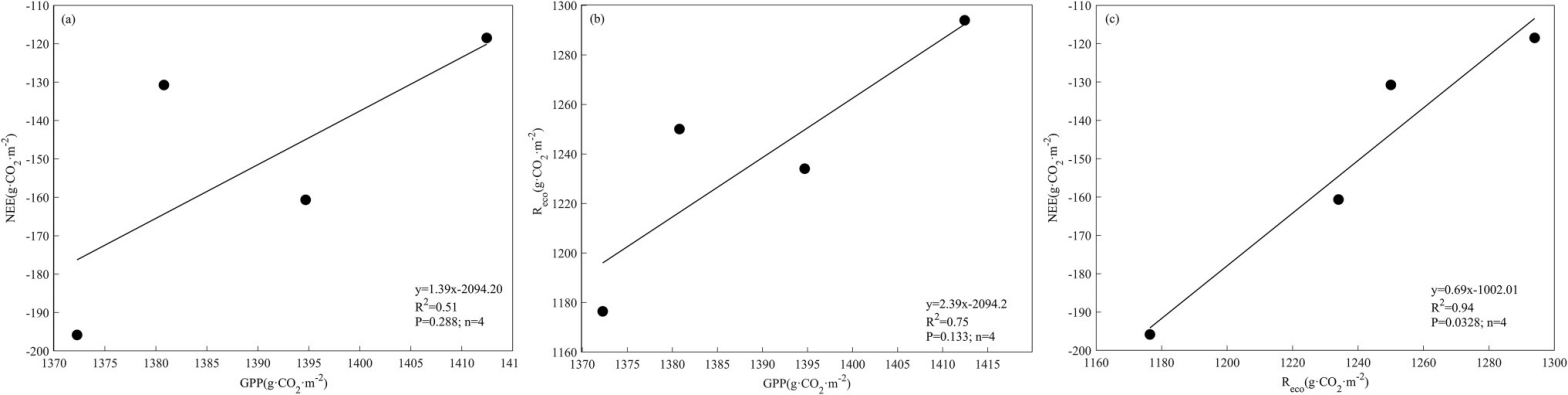
Relationships between net ecosystem exchange of carbon (NEE), ecosystem respiration (R_eco_) and gross primary productivity (GPP) in the alpine meadow.

GPP and R_eco_ are particularly important for net CO_2_ uptake. However, greater GPP may not cause greater net CO_2_ uptake, and greater R_eco_ may not produce lower net CO_2_ uptake or net CO_2_ release (Fig 5a). In this study, the absolute value of NEE in 2011 was greater than that of the other years, but GPP and R_eco_ were lower than those of the other years. The year 2010 had the same GPP as that of 2011, but the absolute value of NEE of 2011 was lower than that of 2010 (Fig 5 and Table 2). There was no significant relationship between NEE and GPP or between R_eco_ and GPP. However, the relationship between NEE and R_eco_ was significant (Fig 5c). These results showed that on an annual scale, the NEE was regulated by R_eco_ and GPP, and R_eco_ was particularly important for the NEE.

**Table 2:** Annual gross primary productivity (GPP, g·CO_2_·m^-2^·yr^-1^) and ecosystem respiration (R_eco_, g·CO_2_·m^-2^ yr^-1^) in alpine meadow.

Hydrological year^a^	GPP	*R*_eco_
2008–2009	1412.45	1293.96
2009–2010	1380.79	1250.04
2010–2011	1372.27	1176.43
2011–2012	1394.69	1234.04
Mean	1390.05	1238.62
STD	17.56	48.597

^a^ Hydrological year starts on 1 October and ends on 30 September of the next year.

### Environmental controls on seasonal and annual carbon fluxes

Environmental factors have important effects on carbon fluxes in terrestrial ecosystems. In the growing season, linear regression revealed significant correlations between the NEE and the Ta, PPT, SWC and PAR. There were significant correlations between R_eco_ and Ta, PPT, SWC, VPD and SWC. There were significant correlations between GPP and Ta, PPT, SWC, SWAC and PAR (Table 3). However, there may be mutually certain relationships and influences in those environmental factors, and the relationship cannot reflect the effect of environmental factors on the CO_2_ fluxes. Thus, partial correlation and stepwise regression analyses were applied to further explore the effects of environmental factors on carbon fluxes. The partial correlation and the stepwise regression analyses showed that the main factor that influenced monthly NEE, R_eco_ and GPP was Ta (Tables 4 and 5).

**Table 3:** Linear regressions between month gross primary production (GPP, g·CO_2_·m^-2^), ecosystem respiration (R_eco_, g·CO_2_·m^-2^), net ecosystem exchange (NEE, g·CO_2_·m^-2^) and monthly environmental factors in the growing season, including air temperature (Ta, °C), precipitation (PPT, mm), soil water content (SWC, cm^3^·cm^−3^), and photosynthetically active radiation (PAR).

Factor	GPP			R_eco_			NEE		
	Linear Equation	R^2^	P	Linear Equation	R^2^	P	Linear Equation	R^2^	P
*T*_a_	**GPP = 58.97Ta-36.75**	**0.89**	**0.00**	**R**_**eco**_ **= 28.59Ta-60.33**	**0.90**	**0.00**	**NEE = -30.39Ta+97.08**	**0.80**	**0.00**
PPT	**GPP = 3.24PPT+56.66**	**0.50**	**0.00**	**R**_**eco**_ **= 1.42PPT+115.76**	**0.42**	**0.00**	**NEE = -1.82PPT+59.10**	**0.54**	**0.00**
VPD	GPP = 1542.80 VPD-162.73	0.16	0.08	**R**_**eco**_ **= 819.80 VPD-120.83**	**0.20**	**0.04**	NEE = -726.05 VPD+141.90	0.12	0.13
SWC	**GPP = 712.76SWC+10.20**	**0.22**	**0.04**	**R**_**eco**_ **= 349.65SWC+81.53**	**0.23**	**0.03**	**NEE = -363.11SWC+71.33**	**0.20**	**0.04**
PAR	**GPP = 4.06PAR-270.24**	**0.24**	**0.03**	R_eco_ = 1.75PAR-23.58	0.19	0.06	**NEE = -2.31AR+246.66**	**0.26**	**0.02**

**Table 4:** Partial correlation coefficients between carbon budgets and site characteristics in the growing season.

CO_2_ fluxes	*T*_a_	PPT	VPD	SWC	PAR
NEE	-0.65**	-0.45	-0.17	0.20	-0.26
R_ceo_	0.80**	0.19	0.19	0.06	0.04
GPP	0.78**	0.40	0.21	0.10	0.20

Notes:

* significant at the 0.05 level;

** significant at the 0.01 level.

**Table 5:** Regression models between carbon budgets and environmental factors in the growing season.

CO_2_ fluxes	*T*_a_	PPT	VPD	SWC	PAR	Intercept	R^2^
NEE	-30.39	-	-	-	-	97.08	0.80
R_ceo_	28.59	-	-	-	-	60.33	0.90
GPP	58.99	-	-	-	-	-36.75	0.89

Annually, linear regression, partial correlation analysis and stepwise regression analysis revealed that there was no significant correlation between NEE, R_eco_, and GPP and environmental factors.

## Discussion

The alpine meadow ecosystem is one of the most important vegetation types worldwide. The study of the carbon fluxes in these ecosystems can improve our understanding of their responses to climate change [42, 43]. Some studies have examined the CO_2_ fluxes of alpine meadow, alpine shrub-meadow, and alpine wetland meadow based on EC measurements on the Tibetan Plateau [8, 17, 44–51] and revealed that some sites were sinks of atmospheric CO_2_ [9, 18, 44–50], while others were CO_2_ sources on an annual scale [18, 51]. On a seasonal scale, in the growing season, the ecosystems were CO_2_ sinks, and in the non-growing season, the ecosystems were CO_2_ sources [9, 44]. Differences occur in the different microclimates, vapor sources of precipitation, underlying surface conditions, vegetation types and influences of human activities, all of which affect the intensity of a carbon source or sink. The results obtained in the present study showed that the alpine meadow ecosystems in Suli were carbon sinks in the growing season and carbon sources in the non-growing season. Because the carbon sink intensity was greater than the carbon source intensity, the alpine meadow ecosystem was a carbon sink at the annual level. The annual CO_2_ sink of alpine meadow in this study was smaller than that of Haibei and Arou, greater than that of Sanjiangyuan, and roughly equal that of Naqu. In addition, the annual CO_2_ sink of alpine meadow in this study was smaller than that of alpine shrub meadow in Haibei, alpine wetland meadow in Yushu, and Qinghai Lake (Table 6).

**Table 6:** Comparison of net ecosystem CO_2_ exchange (NEE) among different grassland ecosystems.

Sit	Ecosystem type	Latitude	Temperature	Precipitation	NEE	Period	Source
Suli, China	Alpine meadow	38°25'	-3.8	364.8	-35.0 to -48.1	2009–2012	This study
Haibei, China	Alpine meadow	37°36'	−1.7	561.0	−193 to −79	2002–2004	Kato et al., 2006
	Alpine shrub-meadow	37°39'	−1.7	570.0	−85 to −52	2004–2005	Fu et al., 2009
	Alpine wetland meadow	37°35'	-1.1	510.4	44.0 to 173.2	2004–2006	Zhao et al., 2010
	Alpine shrubland	37°36'	-1.0	580.0	-132.0 to -10.6	2003–2012	Li et al., 2016
	Alpine meadow	36°57′	0.8	398.2	-74.0	2010–2011	Zhang., 2012
Damxung, China	Alpine meadow	30°10'	1.3	480	37 to 55	2004–2005	Fu et al., 2009
Arou, China	Alpine meadow	38°03'	0.7	400.0	-156	2009	Wang et al, 2014
Yushu, China	Alpine wetland	33°10'	-	-	-126.8	2015	Zhang., 2017
Naqu, China	Alpine meadow	31°37'	-1.3	465.7	-41.3	2008	Zhu et al., 2015
Sanjiangyu, China	Alpine meadow	34°17'	-0.5	500.0	-30.3	2006	Wu et al., 2010
Qinghailake, China	Alpine wetland	36°41'	1.87	282.0	-347.1	2012	Wang, 2015

The GPP and R_eco_ are particularly important for net carbon uptake. Whether GPP or R_eco_ contributes more to the NEE of carbon is still being disputed. Some scholars have shown that the NEE changed with R_eco_ while GPP was constant [52]. Other studies have shown that GPP and R_eco_ are both important for NEE under water deficit conditions [53, 54]. The results obtained in the present study showed that at the annual scale, a higher GPP did not produce increased net carbon uptake. There was no significant correlation between annual GPP, R_eco_ and NEE, while there was a significant correlation between annual NEE and R_eco_. This result indicates that R_eco_ is particularly important for NEE at the annual time scale. This result may occur in the growing season when there was not water stress, as the thawing of permafrost and ample precipitation provide enough water for grass growth. In the non-growing season, cold temperatures wilted plants; thus, the GPP was zero, R_eco_ from soil microorganisms was equal to NEE, and water stress had little effect on R_eco_.

The length of the growing season has an important effect on CO_2_ sequestration [55–58]. Some scholars found that a longer growing season resulted in a greater net CO_2_ uptake [4, 59]. However, others indicated that a longer growing season resulted in a net loss of ecosystem CO_2_ due to longer periods of ecosystem respiration in autumn and less snow water [42, 60–62]. Our data showed that the net CO_2_ uptake increased as the growing season became longer. With a 1-day increase in the growing season, the increased rate of CO_2_ uptake by the ecosystem was 4.4 g·CO_2_·m^2^. This rate is lower than the 7.3 g·CO_2_·m^2^ in savanna, the 21.6 g·CO_2_·m^2^ in temperate deciduous forests and the 25.3 g·CO_2_·m^2^ in boreal aspen forests [63].

Environmental factors have important effects on carbon fluxes in terrestrial ecosystems. Carbon fluxes have been shown to be controlled by climate factors, such as precipitation, temperature, and radiation [64–66]. At mid-to-high latitudes, air temperature is the main factor that influences CO_2_ fluxes. At subtropical and tropical latitudes, radiation and water are the main factors that influence CO_2_ fluxes [67, 68]. In certain regions of Asia, precipitation is more important for CO_2_ fluxes [69]. Kato, Tang (9) showed that the CO_2_ fluxes of alpine meadows are controlled by temperature. This study found that Ta played a critical role in regulating the variations in monthly GPP, R_eco_ and NEE. Temperature is one of the main ecological factors that affects plant growth and development [9, 15, 70]. At certain temperatures, plants can grow normally, and when the cumulative effect of temperature reaches a certain value, plants can complete the growth period. In addition, temperature affects the physiological metabolic process of plants by changing the activity of enzymes [71]. The study area is located in the permafrost area, with extremely low temperatures in the non-growing season. Cold temperatures wilt plants, so water stress has little effect on the carbon fluxes. In the growing season, the permafrost begins to thaw and provide enough water for grass growth. In addition, the precipitation of the growing season was approximately 90% of the total annual rainfall, which meant precipitation provided enough water for grass growth. Therefore, the strong influence of temperature on the carbon fluxes may be related to the reduction in water stress. Thermal conditions are an important factor that control plant photosynthesis in a temperature-limited environment [72]. On an annual scale, linear regression, partial correlation analysis and stepwise regression analysis revealed that there was no significant correlation between NEE, R_eco_, and GPP and environmental factors. Some studies have also shown that non-climate factors are a major factor causing carbon flux variability [73–75]. This result may be because the changes in T_a_, T_s5_ cm, VPD, R_n_, SWC and PAR are small at the annual level, but they are greatly different in the growing season and non-growing season. In addition, the uncertainty from gap filling and instrument error and the uncertainty due to spatial variability contribute more uncertainty in annual carbon flux estimates [73].

## Conclusions

The CO_2_ fluxes measured with the EC technique in the alpine meadow ecosystem on the northeastern QTP from 2009 to 2012 showed that the alpine meadow acted as a carbon sink. Ecosystem respiration is particularly important for net carbon uptake. A longer growing season was attributed to an increase in C sequestration. In the study area, we found that temperature was the dominant factor influencing the seasonal variation in CO_2_ fluxes and that there was no dominant factor influencing the inter-annual variations in CO_2_ fluxes.

## Supporting information

S1 Data The data used in the article. (XLSX)
